# VP39 of *Spodoptera litura* multicapsid nucleopolyhedrovirus cannot efficiently rescue the nucleocapsid assembly of *vp39*-null *Autographa californica* multiple nucleopolyhedrovirus

**DOI:** 10.1186/s12985-021-01553-9

**Published:** 2021-04-20

**Authors:** Sainan Li, Bingming Ou, Yina Lv, Tian Gan, Haizhou Zhao, Wenhua Liu

**Affiliations:** Department of Biology, Zhaoqing University, Zhaoqing, 526061 China

**Keywords:** AcMNPV, *vp39*, Nucleocapsid assembly, Viral DNA packaging, SpltMNPV

## Abstract

**Background:**

*Autographa californica* multiple nucleopolyhedrovirus (AcMNPV) *vp39* is conserved in all sequenced baculovirus genomes. In previous studies, VP39 has been identified as the major capsid structure protein of baculoviruses and found to be essential for nucleocapsid assembly. The nucleocapsid composition and structure of Group I and II NPVs of the *Alphabaculovirus* genus are very similar. It is not clear whether the major capsid structure protein VP39 of Group I NPVs is functionally identical to or substitutable with the Group II NPV VP39. In this study, the function of Group II *Spodoptera litura* MNPV (SpltMNPV) VP39 in Group I AcMNPV was characterized.

**Methods:**

Sequence alignment of AcMNPV VP39 and SpltMNPV VP39 was performed using Clustal X and edited with GeneDoc. To determine whether VP39 of Group I NPVs can be functionally substituted by Group II NPV VP39, a *vp39*-null AcMNPV (vAcvp39KO) and a *vp39*-pseudotyped AcMNPV (vAcSpltvp39:FLAG), in which the Group I AcMNPV *vp39* coding sequence was replaced with that of SpltMNPV from Group II NPVs, were constructed via homologous recombination in *Escherichia coli*. Using an anti-FLAG monoclonal antibody, immunoblot analysis was performed to examine SpltMNPV VP39 expression. Fluorescence and light microscopy were used to monitor viral replication and infection. Viral growth curve analysis was performed using a fifty percent tissue culture infective dose (TCID_50_) endpoint dilution assay. Viral morphogenesis was detected using an electron microscope.

**Results:**

Sequence alignment indicated that the N-termini of AcMNPV VP39 and SpltMNPV VP39 are relatively conserved, whereas the C-terminus of SpltMNPV VP39 lacks the domain of amino acid residues 306–334 homologous to AcMNPV VP39. Immunoblot analysis showed that SpltMNPV VP39 was expressed in vAcSpltvp39:FLAG. Fluorescence and light microscopy showed that vAcSpltvp39:FLAG did not spread by infection. Viral growth curve analysis confirmed a defect in infectious budded virion production. Electron microscopy revealed that although masses of abnormally elongated empty capsid structures existed inside the nuclei of Sf9 cells transfected with vAcSpltvp39:FLAG, no nucleocapsids were observed.

**Conclusion:**

Altogether, our results demonstrated that VP39 from SpltMNPV cannot efficiently substitute AcMNPV VP39 during nucleocapsid assembly in AcMNPV.

## Main text

*Baculoviridae* is a family of enveloped, rod-shaped, large, circular, double-stranded DNA viruses [1]. These viruses are composed of four genera: *Alphabaculovirus*, *Betabaculovirus*, *Gammabaculovirus*, and *Deltabaculovirus* [2]. The members of the *Alphabaculovirus* genus can be further divided into Group I and II nucleopolyhedroviruses (NPVs) [3]. Taxonomically, *Autographa californica* multiple NPV (AcMNPV) is the prototype of the *Baculoviridae* family. Two different forms of virions, namely, budded virions (BVs) and occlusion-derived virions (ODVs), are produced during the biphasic replication cycle of baculoviruses. BVs are required for spreading infections among susceptible insect cells and tissues, resulting in systemic infection. ODVs initiate primary infection in a susceptible host and are responsible for transmitting infections among insect hosts [4]. The main difference between BVs and ODVs is the composition and origin of their envelopes, whereas their nucleocapsid structures appear to be similar and are composed of a nucleoprotein core and a cylindrical capsid sheath. Viral DNA is synthesized in the virogenic stroma (VS) and packaged into capsids to form nucleocapsids, and capsid assembly seems to be independent of viral DNA packaging [5].

VP39, the major capsid structure protein, is arranged in stacked rings around the nucleoprotein core with monomers [6, 7]. The AcMNPV *vp39* (*orf89*) gene is located between AcMNPV nucleotides (nt) 75,534 and 76,577 [8]. Homologs of AcMNPV *vp39* are present in all sequenced baculovirus genomes. Deletion of *vp39* from *Bombyx mori* NPV (BmNPV) resulted in no obvious BV production [9]. Recently, in a study reported by Bai et al., deletion of AcMNPV *vp39* led to a defect in nucleocapsid assembly [10]. The conserved glycine residue 276 plays an important role in the BmNPV VP39 function, including the structural assembly of capsids and viral DNA packaging [11].

**Fig. 1:**
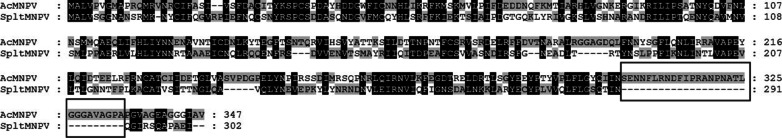
AcMNPV VP39 and SpltMNPV VP39 sequences were aligned using Clustal X and edited with GeneDoc. The following sequences were used: NP_054119.1 for AcMNPV and NP_258349.1 for SpltMNPV. Amino acids with black, dark grey, or light grey shading denote 100%, 80%, or 60% conservation in two sequences, respectively. The box indicates the location of the nonconservative amino acid region in the C-terminus

The nucleocapsid composition and structure are very similar between Group I and II NPVs of the *Alphabaculovirus* genus, whereas their gene content and BV fusion protein are significantly different [1, 12]. It is not clear whether the major capsid structure protein VP39 of Group I NPVs is functionally identical to or substitutable with the Group II NPV VP39. The amino acid sequence identity of VP39 ranges from 35.28% to 51.32% between Group I and Group II NPVs and ranges from 37.22% to 45.71% between AcMNPV and Group II NPVs. The overall amino acid sequence identity between AcMNPV VP39 and SpltMNPV VP39 is 37.22% [13]. Sequences of AcMNPV VP39 and SpltMNPV VP39 were aligned and edited using Clustal X [14] and GeneDoc [15], respectively. As shown in Fig. 1, AcMNPV VP39 and SpltMNPV VP39 are relatively conserved in the N-terminus, whereas SpltMNPV VP39 lacks a domain of amino acid residues 306–334 homologous to AcMNPV VP39 in the C-terminus. To determine whether the major capsid structure protein VP39 in Group I AcMNPV can be functionally substituted with Group II SpltMNPV VP39, we produced a recombinant AcMNPV (vAcSpltvp39:FLAG), in which AcMNPV *vp39* was replaced with SpltMNPV *vp39.*

**Fig. 2:**
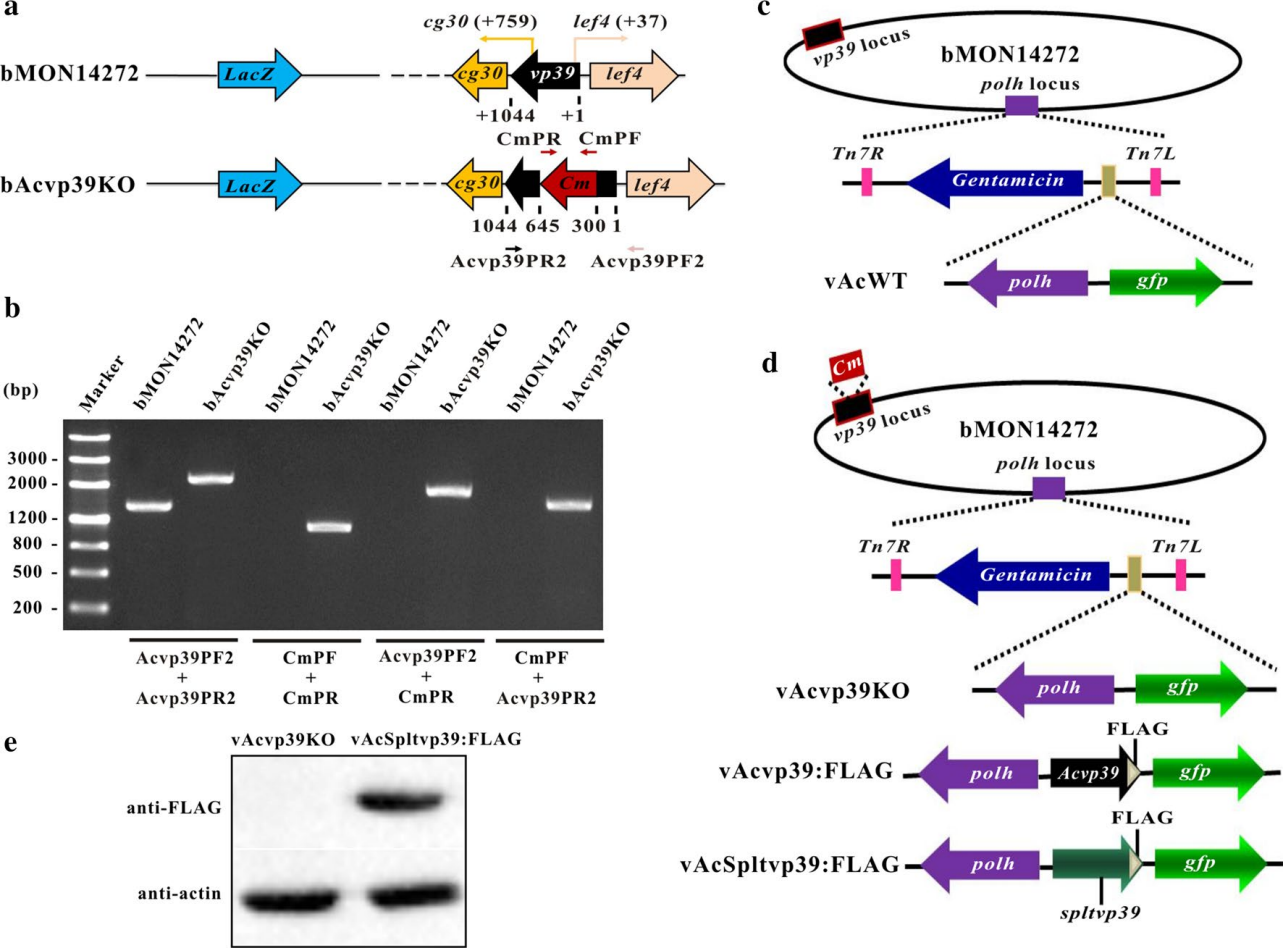
Construction of recombinant viruses and their confirmation. (**a**) Strategy for bAcvp39KO construction. AcMNPV *vp39* ORF has 1044 nt. The first nucleotide of the initiation codon ATG of *vp39* is indicated as + 1. The 5′ late transcription start site of *cg30* is located 759 nt downstream of the initiation codon ATG of *vp39*, and the 5′ transcription start site of *lef4* is located 37 nt downstream of the initiation codon ATG of *vp39*. The 344-bp fragment between 300 and 645 nt downstream of *vp39* ATG in the bMON14272 genome was replaced with a 1038-bp *Cm* cassette via ET homologous recombination to generate the *vp39* deletion bacmid, bAcvp39KO. (**b**) PCR confirmation of bacmids. Primer pairs used in this study are shown below. Bacmid DNA templates are shown above each lane, and the migration of DNA markers is shown to the left in base pairs (bp). (**c**) Schematic diagram of wild-type control virus vAcWT, which was generated by inserting *polh* and *gfp* genes into the *polh* locus of bMON14272 via Tn7-mediated transposition. (**d**) Schematic diagram of vAcvp39KO, vAcvp39:FLAG, and vAcSpltvp39:FLAG. The *vp39* deletion virus, vAcvp39KO, was constructed by inserting *polh* and *gfp* genes into the *polh* locus of bAcvp39KO. The AcMNPV *vp39* (*Acvp39*) gene under the control of its native promoter with a FLAG tag (indicated as a grey triangle) prior to the *vp39* stop codon together with the *polh* and *gfp* genes were inserted into the *polh* locus of bAcvp39KO to construct the rescue virus, vAcvp39:FLAG. The SpltMNPV *vp39* (*Spltvp39*) gene under the AcMNPV *vp39* promoter control with a FLAG tag (indicated as a grey triangle) prior to the SpltMNPV *vp39* stop codon together with the *polh* and *gfp* genes were inserted into the *polh* locus of bAcvp39KO to construct the *vp39*-pseudotyped virus, vAcSpltvp39:FLAG. (**e**) Sf9 cells were transfected with vAcvp39KO or vAcSpltvp39:FLAG and harvested at 72 h p.t. FLAG-tagged SpltMNPV VP39 or actin was detected by immunoblotting with anti-FLAG or anti-actin (endogenous control) monoclonal antibodies, respectively

To determine whether SpltMNPV VP39 could function in AcMNPV, the bMON14272 bacmid was employed to generate a *vp39*-knockout AcMNPV bacmid (bAcvp39KO) via the λ Red recombination system. According to AcMNPV transcriptomics [16], the late transcription start site of *cg30* and early transcription start site of *lef4* were located 759 nt and 37 nt downstream of the initiation codon ATG of *vp39,* respectively. In the bAcvp39KO bacmid, a 344-bp region of *vp39* (AcMNPV nt 75,934–76,277, between 300 and 645 nt downstream of the *vp39* ATG) was replaced with a 1038-bp *chloramphenicol resistance* (*Cm*) gene cassette, with 300 nt of the 5′-end and 400 nt of the 3′-end of the *vp39* ORF retained to avoid any potential effects on *cg30* and *lef4* (Fig. 2a). The absence of *vp39* and replacement with the *Cm* gene in the AcMNPV bacmid were confirmed by PCR analysis. As shown in Fig. 2b, primers Acvp39PF2 (5′-GAGCTCCAAATTTGATTTCAATTTTATCGTGTTGGT-3′)/Acvp39PR2 (5′-GGATCCTTACTTATCGTCGTCATCCTTGTAATCGACGGCTATTCCTCCACCTGCTTC-3′) produced a 1468-bp PCR product in AcMNPV bacmid bMON14272 and a 2162-bp fragment in bAcvp39KO. Similarly, primers CmPF (5′-CCCTTTCGTCTTCGAATAAATACCT-3′)/CmPR (5′-TAAACCAGCAATAGACATAAGCGGC-3′), Acvp39PF2/CmPR, and CmPF/Acvp39PR2 produced no PCR product in AcMNPV bacmid bMON14272, but 1038-, 1738-, and 1438-bp fragments were amplified in bAcvp39KO, respectively. These results demonstrated that the *Cm* gene successfully replaced the target deletion region of *vp39*. The SpltMNPV *vp39* gene (SpltMNPV nt 77,450–78,358) under the control of the AcMNPV *vp39* promoter (AcMNPV nt 76,578–76,977) with a FLAG tag prior to the SpltMNPV *vp39* stop codon, together with the *enhanced green fluorescence protein* (*egfp*; referred to as *gfp* in the present study) gene and the AcMNPV *polyhedrin* (*polh*) gene, which were driven by the AcMNPV *ie1* promoter and its own promoter, respectively, were inserted into the *polh* locus of bAcvp39KO to construct the *vp39*-pseudotyped AcMNPV, vAcSpltvp39:FLAG (Fig. 2d). A *vp39*-knockout virus, vAcvp39KO, was used as a negative control and was constructed by inserting the *polh* and *gfp* genes into the *polh* locus of bAcvp39KO (Fig. 2d). Similarly, a wild-type AcMNPV, vAcWT, was used as a positive control and was generated by inserting the two genes into bMON14272 (Fig. 2c). A rescue virus, vAcvp39:FLAG, was constructed to confirm that the phenotype observed in vAcvp39KO resulted from the deletion of *vp39* by inserting the *vp39* gene under the control of its native promoter with a FLAG tag prior to the *vp39* stop codon along with the *polh* and *gfp* genes into the *polh* locus of bAcvp39KO (Fig. 2d). To demonstrate that SpltMNPV VP39 could be expressed in vAcSpltvp39:FLAG, vAcvp39KO or vAcSpltvp39:FLAG bacmid DNA was transfected into Sf9 cells using Cellfectin and harvested at 72 h post transfection (p.t.). Using an anti-actin or anti-FLAG monoclonal antibody, western blotting was performed to detect actin (endogenous control) or SpltMNPV VP39 expression, respectively. SpltMNPV VP39 was detected in Sf9 cells transfected with vAcSpltvp39:FLAG but not in Sf9 cells transfected with vAcvp39KO (Fig. 2e).

**Fig. 3:**
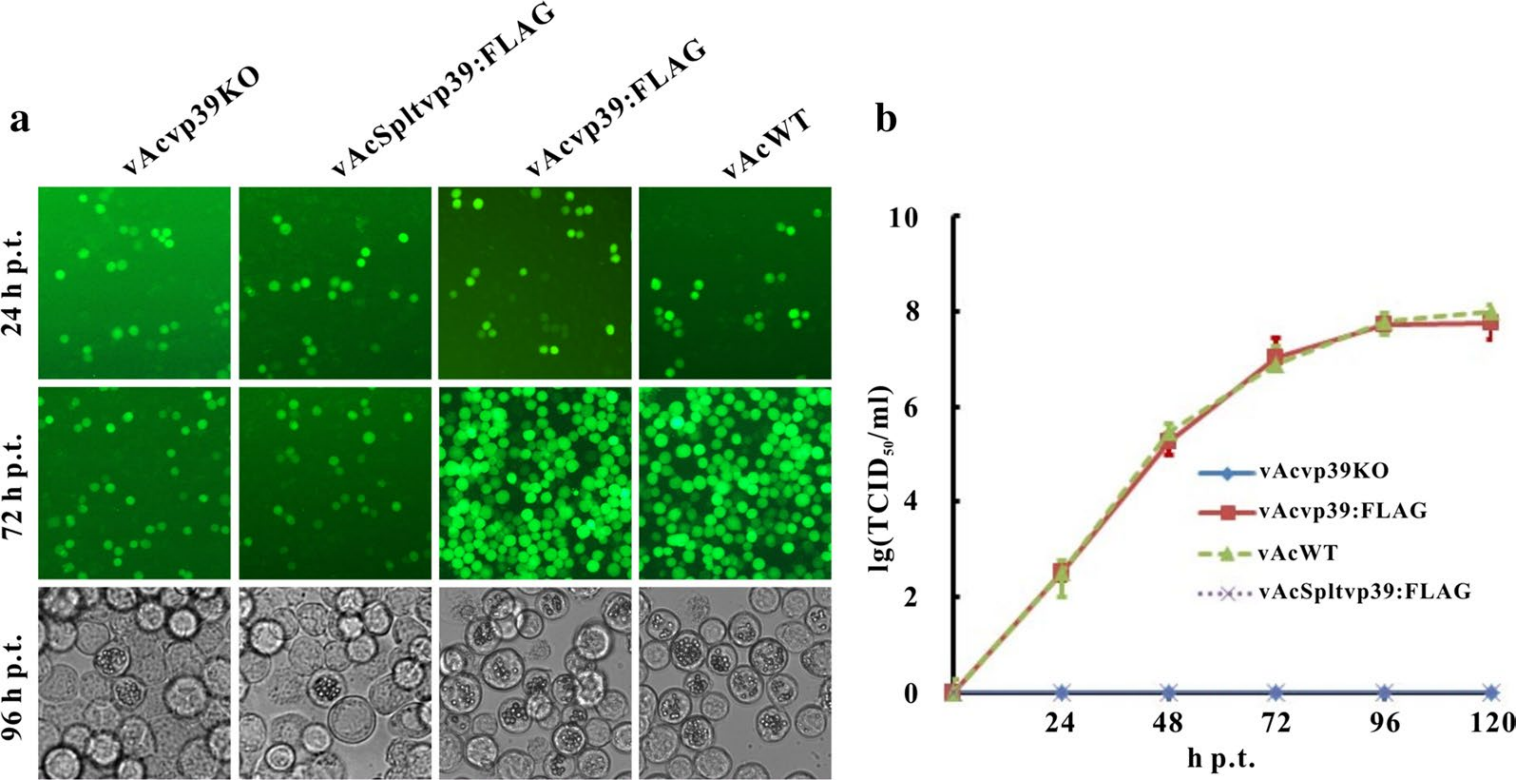
Analysis of viral replication. (**a**) Sf9 cells were transfected with vAcvp39KO, vAcSpltvp39:FLAG, vAcvp39:FLAG, or vAcWT bacmid DNA. Cells were observed using a fluorescence microscope at 24 and 72 h p.t. and a light microscope at 96 h p.t. (**b**) Sf9 cells were transfected with vAcvp39KO, vAcSpltvp39:FLAG, vAcvp39:FLAG, or vAcWT bacmid DNA. At designated time points, supernatants were harvested, and BV titers were determined using TCID_50_ endpoint dilution assays. Each value represents the average titer of three independent assays; error bars represent standard deviations

To investigate the effect of *vp39* replacement on viral replication, vAcvp39KO, vAcSpltvp39:FLAG, vAcvp39:FLAG, or vAcWT bacmid DNA was transfected into Sf9 cells, and viral replication and infection were monitored by fluorescence microscopy and light microscopy. At 24 h p.t., no obvious differences in the amounts of GFP-positive cells were observed among all four groups, indicating comparable transfection efficiencies (Fig. 3a). As expected, almost all Sf9 cells transfected with vAcWT or vAcvp39:FLAG bacmid DNA showed GFP fluorescence at 72 h p.t., and the number of fluorescent cells exhibited no detectable increase from 24 to 72 h p.t. in the vAcvp39KO-transfected cells. Notably, in vAcSpltvp39:FLAG-transfected cells, the variation in the number of fluorescent cells from 24 to 72 h p.t. was the same as that of vAcvp39KO, showing the inability of *vp39*-pseudotyped AcMNPV to produce infectious BVs to infect adjacent cells (Fig. 3a). In the four groups, occlusion body (OB) formation was observed using a light microscope (Fig. 3a), indicating that the infection progression among the four viruses was similar. Using virus supernatants harvested from vAcvp39KO-, vAcSpltvp39:FLAG-, vAcvp39:FLAG-, or vAcWT-transfected cells, viral growth curve analysis was performed using a fifty percent tissue culture infective dose (TCID_50_) endpoint dilution assay to further evaluate the ability of vAcSpltvp39:FLAG to produce infectious BV. No significant difference in the ability to generate infectious BVs was noted between vAcvp39:FLAG and vAcWT, whereas the infectious BVs of vAcvp39KO or vAcSpltvp39:FLAG were undetectable at any time point, even up to 120 h p.t. (Fig. 3b). These results showed that SpltMNPV VP39 cannot rescue the infectious BV production of *vp39*-deleted AcMNPV.

**Fig. 4:**
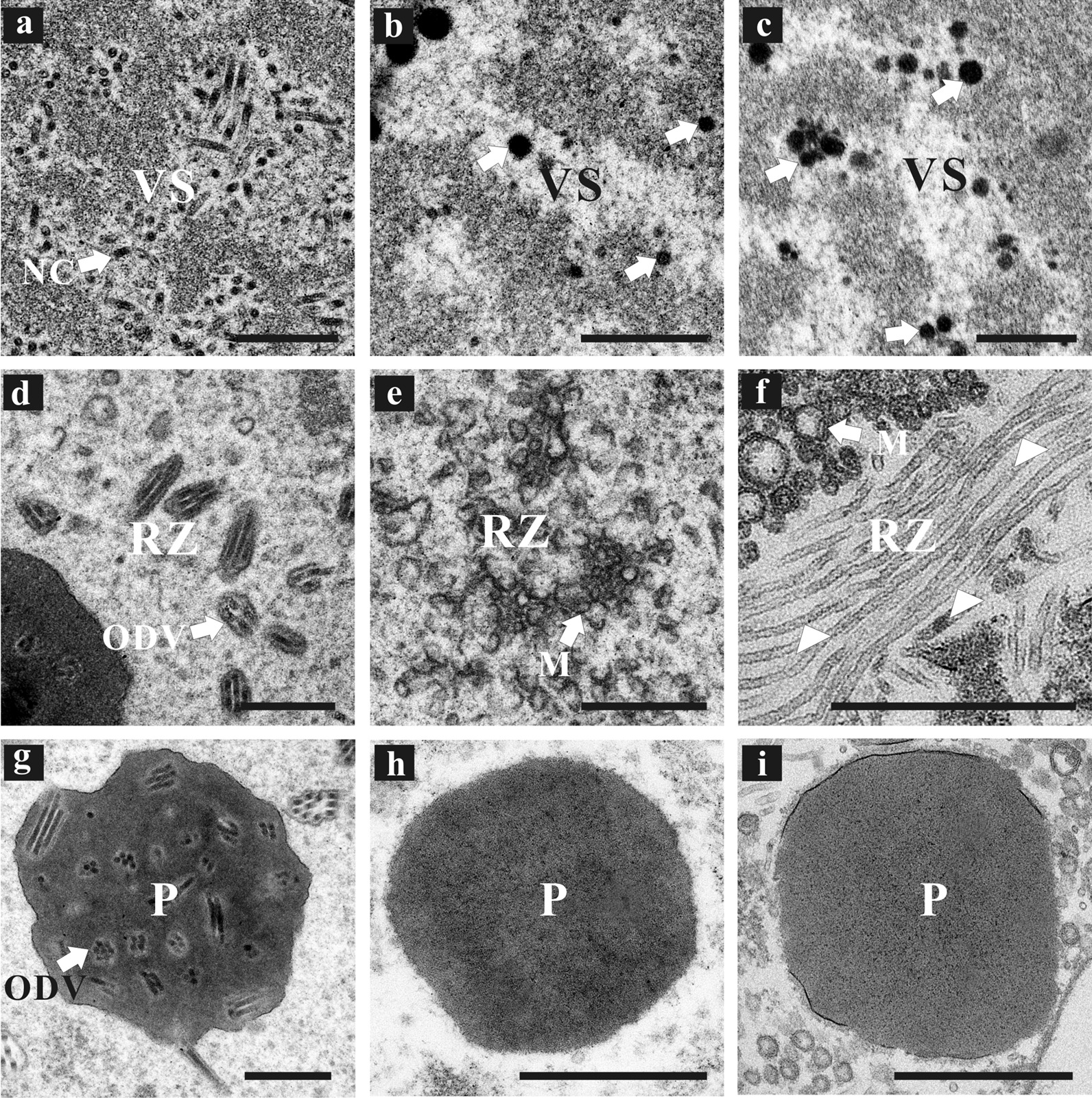
Electron microscopy of Sf9 cells transfected with vAcvp39:FLAG (**a**, **d**, and **g**), vAcvp39KO (**b**, **e**, and **h**), or vAcSpltvp39:FLAG (**c**, **f**, and **i**) at 72 h p.t. (**a**) Portion of virogenic stroma (VS) showing nucleocapsids with viral DNA. (**b**) and (**c**) Portion of VS without nucleocapsids, exhibiting abnormal electron-dense bodies (white arrows). (**d**) Portion of the ring zone with occlusion-derived virions (ODVs). (**e**) Portion of the ring zone without ODV, showing numerous microvesicles. (**f**) Portion of the ring zone without ODV, showing numerous microvesicles and masses of abnormally long electron-lucent capsid-like structures lacking viral DNA (white triangles). (**g**) Magnified view of occlusion body (OB) embedding with ODVs. (**h**) and (**i**) Magnified view of OB without ODVs. NC, nucleocapsid; RZ, ring zone; M, microvesicle; P, OB. Scale bar, 500 nm

Thin sections of vAcvp39KO-, vAcSpltvp39:FLAG-, vAcvp39:FLAG-, and vAcWT-transfected Sf9 cells at 72 h p.t. were observed using an electron microscope to further investigate whether the replacement of *vp39* interferes with virus morphogenesis (Fig. 4). Sf9 cells transfected with vAcWT (data not shown) or vAcvp39:FLAG showed typical characteristics of baculovirus infection, including a well-defined VS containing numerous rod-shaped, electron-dense nucleocapsids formed in the nuclei (Fig. 4a), numerous virus-induced intranuclear microvesicles emerged in the ring zone (data not shown), a large number of ODVs containing nucleocapsids (Fig. 4d) and developing OBs embedding numerous ODVs existed in the ring zone (Fig. 4g). In cells transfected with vAcvp39KO, although morphologically indistinguishable VS was observed in the nuclei (Fig. 4b), no capsid structures emerged in the VS (Fig. 4b) and ring zone (Fig. 4e). A large number of abnormal electron-dense bodies, which typically exist in AcMNPV-infected cells with anomalous nucleocapsids [17, 18], appeared in the VS (Fig. 4b). Numerous virus-induced intranuclear microvesicles existed in the ring zone (Fig. 4e), but no ODVs were observed in the ring zone (Fig. 4e) or the OBs (Fig. 4h). Similar to vAcvp39KO, in all vAcSpltvp39:FLAG-transfected Sf9 cells, a typical VS without nucleocapsids but with a large number of electron-dense bodies was observed in the nuclei (Fig. 4c), numerous virus-induced intranuclear microvesicles emerged in the ring zone (Fig. 4f), and no ODVs existed in the ring zone (Fig. 4f) or the OBs (Fig. 4i). However, interestingly, in vAcSpltvp39:FLAG-transfected Sf9 cells, many abnormally long electron-lucent capsid-like structures lacking an electron-dense core indicative of nucleoprotein that contains viral DNA were observed in the nuclei (Fig. 4f). These results demonstrated that SpltMNPV VP39 has the ability to assemble tubular capsid-like structures but not nucleocapsids in AcMNPV.

Similar to vAcSpltvp39:FLAG, the phenotypes of a series of nucleocapsid-associated gene individual deletion AcMNPV, such as *pk-1*, *ac53*, *vp1054*, *vlf-1*, *vp91*, *38 k*, *p6.9*, *BV/ODV-C42*, and *ac102*, included the cessation of nucleocapsid assembly and the appearance of a large number of long empty capsid-like structures in the nucleus [17–25]. This finding emphasizes the need for further research to completely elucidate the mechanism of nucleocapsid assembly. Among the proteins encoded by these genes, P6.9 can bind baculoviral DNA and is responsible for baculoviral DNA condensation for packaging into capsids to form nucleocapsids [26, 27], and 38 K is critical for P6.9 to perform this function by dephosphorylating it [28].

Nucleocapsid assembly is a very complex process that demands numerous proteins to be involved in coordinatively. Previous studies showed that VP39 can interact with itself and other nucleocapsid-associated proteins and cellular proteins, including 38 K, ODV-EC27, BV/ODV-C42, P78/83, and actin [29–32], implying that these proteins may be jointly involved in crucial steps in nucleocapsid assembly.

Once proteins interact with each other, allosteric transition may confer a structure that differs from that noted for individual proteins [33]. Here, we report that replacing AcMNPV *vp39* with SpltMNPV *vp39* resulted in interruption of nucleocapsid assembly, although a number of long capsid structures lacking viral DNA appeared in the nucleus. Considering that SpltMNPV VP39 lacks the homologous sequence of a C-terminal domain in AcMNPV VP39 (Fig. 1), which may lead to the structural difference between SpltMNPV VP39 and AcMNPV VP39, it is possible that, in the case of vAcSpltvp39:FLAG, SpltMNPV VP39 cannot appropriately interact with 38 K, ODV-EC27, BV/ODV-C42, P78/83, actin or other related proteins, or that the interactions are weak, which may affect the allosteric transition of these proteins. The incomplete transition might not allow proteins to be completely functional; for instance, the decreased activity of 38 K may not be enough to properly mediate the dephosphorylation of P6.9, thus resulting in no viral DNA packaging into capsids to form normal nucleocapsids in vAcSpltvp39:FLAG. Further studies are required to understand the details of this process.

## Conclusions

In summary, our study showed that SpltMNPV *vp*39 substitution in AcMNPV interrupted the assembly of nucleocapsids, resulting in no BV or ODV being produced. To the authors’ knowledge, this is the first study reporting on the function of Group II SpltMNPV VP39 in Group I AcMNPV. Further studies are needed to disclose the molecular mechanisms of nucleocapsid assembly. These studies will likely provide information on the evolution of genes to be adapted for each virus-host interaction to facilitate virus replication and amplification in the host.

## Data Availability

All data generated or analysed during this study are included in this published article.

## Abbreviations

AcMNPV: *Autographa californica* multiple nucleopolyhedrovirus
SpltMNPV: *Spodoptera litura* MNPV
BmNPV: *Bombyx mori* NPV
TCID_50_: Fifty percent tissue culture infective dose
BV: Budded virion
ODV: Occlusion-derived virion
VS: Virogenic stroma
nt: Nucleotide
bp: Base pair
*Cm*: *Chloramphenicol resistant*
*gfp*: *Enhanced green fluorescence protein*
*polh*: *polyhedrin*
OB: Occlusion body
p.t.: Post transfection
